# The Impact of Beehive Proximity, Human Activity and Agricultural Intensity on Diptera Diversity in a Mediterranean Mosaic of Agroecosystems, with a Focus on Pest Species

**DOI:** 10.3390/ani13061024

**Published:** 2023-03-10

**Authors:** Barbara Sladonja, Ivana Tlak Gajger, Mirela Uzelac, Danijela Poljuha, Clara Garau, Nediljko Landeka, Miroslav Barták, Giovanni Bacaro

**Affiliations:** 1 Institute of Agriculture and Tourism, Karla Huguesa 8, 52440 Poreč, Croatia; 2 Faculty of Veterinary Medicine, University of Zagreb, Heinzelova 55, 10000 Zagreb, Croatia; 3 Department of Life and Environment Botanical Section, University of Cagliari, Viale S. Ignazio da Laconi 13, 09123 Cagliari, Italy; 4 Public Health Institute of the Istrian Region, Nazorova 23, 52100 Pula, Croatia; 5 Department of Zoology and Fisheries, Faculty of Agrobiology, Food and Natural Resources, Czech University of Life Sciences Prague, Kamýcká 129, 165 00 Praha 6, Suchdol, Czech Republic; 6 Department of Life Sciences, University of Trieste, Via L. Giorgieri 10, 34127 Trieste, Italy

**Keywords:** anthropic parameters, biodiversity preservation, composition, invasive species, inventory, mosaic ecosystems, new records, pest, richness

## Abstract

**Simple Summary:**

Diptera are rich in species, contribute significantly to plant diversity, and have the potential to assess habitat health. They also include many agricultural and forestry pests. Our study provides baseline information on Diptera and Vespidae diversity in the Mediterranean mosaic of agroecosystems. Additionally, we summarized the information on the importance of human influence on Diptera diversity. We carried out an inventory of Diptera in Croatia using a set of traps placed in the proximity of honeybee hives. We determined the presence of pests and newly introduced species. A total of 94 species belonging to 24 families were recorded, including 7 important agricultural and forest pests of Diptera and 17 new records for Croatia. Results pointed out that total insect species richness, pest species richness, and the first findings depend on human activities. Furthermore, the number of honeybee colonies was negatively correlated with total species richness, while anthropogenic influence was positively correlated with pest species richness.

**Abstract:**

Diptera, with their participation in pollination, significantly contribute to the maintenance of plant diversity, and they also have great potential for assessing habitat health and preserving it. A decline in their abundance and diversity has been recorded worldwide as a consequence of biotic, abiotic, and anthropic alterations. In addition to pollinators, these orders include agricultural and forestry pests, which are a threat to both cultivated and wild plants that are very important to the economy. Many pests have escaped from their native areas, and it is important to monitor their spread to implement sustainable means of control. Our study provides baseline information on Diptera and Vespidae diversity in the Mediterranean mosaic of agroecosystems, giving information on the importance of human influence on insect diversity. We carried out an insect inventory in Istria, Croatia, using a set of traps placed in the proximity of beehives. This study was also important in determining the presence of pests and newly introduced species. A total of 94 species from 24 families were recorded—7 important agricultural pests of Diptera and 17 new records for Croatia. The correlation between species diversity and environmental and anthropogenic factors leads to the conclusion that total insect species richness, pest species richness, and the first findings depend on human activities. The number of honeybee colonies negatively correlated with species richness, while anthropic influence positively affected total and pest species richness.

## 1. Introduction

Efforts are continuously carried out to measure, estimate, and conserve the diversity of insects in different habitats [1]. Most insects provide many direct (pollination, removal of carcasses and faeces, insect predators and parasites of herbivores including pests), indirect (apian products) services for humans, as well as other important benefits like silk (silk moths), dyes and shellac (scale insects), and tannic acid and ink (insect galls) [2]. Diptera is a diverse insect order not only in species richness but also its structure, habitat needs, ecology, and human interactions [3].

The Mediterranean basin is known as a biodiversity hotspot because it has a lot of different kinds of plants and animals that pollinate them [4]. Pollinators’ decline and loss of biodiversity, directly affecting the worldwide provision of pollination services, have been reported in response to anthropogenic-driven activities [5,6]. Human impact has caused degradation, destruction, and fragmentation of natural habitats, altering pollinator communities [7]. On the other hand, some semi-natural habitats provide good conditions for insects [8].

Maintaining biodiversity in human-dominated landscapes, especially in agriculture-dominated ones, is a topic of great interest. Agriculture intensification and homogeneous agricultural landscapes are known to affect biodiversity negatively [9,10,11]. In the Mediterranean area, agriculture is fragmented into small patches, forming a mosaic landscape [12,13]. The mosaic landscape is defined as structurally complex spaces of variable scale that accommodate different interacting land-cover types of both natural and anthropogenic origin [14]. These traditional Mediterranean multifunctional landscapes [15] are also typical for the Croatian coast.

Croatian agriculture in the last 30 years has been characterized by negative trends both in the use of agricultural land and in the decrease in the economy and the number of producers. The extensive development of tertiary activities such as tourism, with the involvement of most of the working population, is advocated as the leading cause of the decline [16]. Istrian agricultural landscapes are characterized by fragmented parcels, diverse crop production, and mosaic agricultural practice. It is a patchwork of vineyards, olive fields, extensive agriculture, natural karstic vegetation, and semi-natural habitats, making suitable habitats for insect populations. The average agricultural parcel in Istrian County has an area of only 0.51 ha [17]. Goethe et al. (2021) studied the influence of different agricultural landscapes on biodiversity and concluded that agricultural landscapes dominated by wheat are associated with decreased pest abundance in the investigated area [18]. Other authors have also highlighted a need for studying how strategically managed agroecosystems can provide habitats to maximize the conservation of insect taxa [19,20,21,22,23].

Besides their positive role in the ecosystem, insects can also act as pests, depending on the environmental conditions and human activities. Invasive insect pests are a growing problem, and many researchers are working on listing and describing their effects on natural and anthropogenic environments [24]. Of all the insects in the world, only 1% are pests [25], but they are responsible for the loss of 13% of agricultural crops and 9% of forest production [26]. Human activities have significantly increased the dispersal of pests worldwide [24]. More than 200 species of Diptera are considered pests infesting ripening fruits [27,28,29]. Alien species of Diptera have exponentially increased in Europe since the second half of the 20th century [30]. Most alien pests in Europe were introduced unintentionally, and almost one-third originate from North America. Many are important horticulture pests [31]. Fruit flies can cause substantial economic losses worldwide to fruit and vegetable growing and beekeeping [32,33,34].

Honeybee apiaries can influence the diversity and abundance of insect pollinators. To our knowledge, little is known about this topic. There are not many studies about how mosaic landscapes affect the number and types of pollinators, especially in the Mediterranean area. Apiary spots could be a powerful tool for choosing points for efficient pollinator species inventories and monitoring invasive insects and pests. Habitats close to honeybee hives are characterized by plants often visited by honeybees and offer favorable conditions for pollinator species due to the vital number of floral resources. Such conditions could favor the establishment of insect pollinator species but also attract newcomers such as invasive species and pests. Apiary management practices do not affect vegetation structure and diversity but may affect the diversity of certain insect groups. On the other hand, Valido et al. (2019) showed that the expansion of beekeeping affects mutualistic interactions, potentially disturbing the structure and functioning of pollination networks in natural ecosystems and thus negatively impacting the biodiversity of wild pollinators [35]. It was also assessed that numerous honeybee colonies could strongly affect the foraging activity of wild bees in the Mediterranean area [4].

Therefore, this study aims to assess the richness and composition of Diptera and Vespidae in the Istrian region (Croatia) and to perform an inventory of invasive pests in the proximity of honeybee apiaries. Our specific objectives were to: (1) quantify Diptera diversity within 16 sites in proximity of the honeybee hives placed in the mosaic landscape; (2) provide new insights into the distribution of agricultural and forest pests; and (3) Assess the correlation of Diptera and Vespidae diversity with environmental and anthropogenic variables.

## 2. Materials and Methods

### 2.1. Study Area

**Figure 1:**
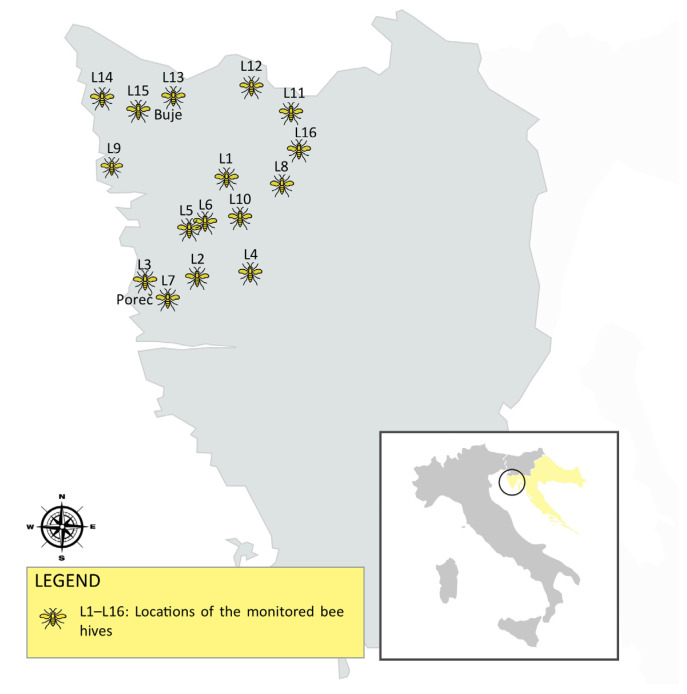
Location of study sites in the Northern Istrian peninsula, Croatia (Poreč and Buje municipalities).

The study area is a typical mosaic of agricultural habitats with fragmented small parcels. Sixteen local beekeepers were randomly selected in the geographical area of the Istrian peninsula (Croatia) between the municipalities of Poreč and Buje (45° N, 13° E) (Figure 1). The size of apiaries ranged from 8 to 90 honeybee colonies (Table S1), which is in line with the Croatian average described for 2018 (57) and twice as large as the European average (22) [36]. A detailed description of each location (coordinates, anthropological and ecological data) is presented in Table S1. The surrounding vegetation is represented by Mediterranean trees (*Quercus* spp., *Quercus pubescens* Willd., *Pinus halepensis* Miller, *Olea europaea* L., *Aesculus hippocastanum* L., *Tilia* sp., *Pinus nigra* Arnold and *Populus* sp.), shrubs (*Carpinus betulus* L., *Rubus* spp., *Cornus mas* L., *Lonicera* spp., *Rhamnus alaternus* L., *Pistacia lentiscus* L., *Hedera helix* L., *Vitis vinifera* L., *Juniperus oxycedrus* L., *Rosmarinus officinalis* L.), invasive alien tree *Robinia pseudoacacia* L., and herbaceous species (*Sorghum* sp., *Trifolium* sp.).

The surveys comprised abundance records of all vascular plant species according to the Braun-Blanquet scale in three vegetation layers (herbaceous: 0–1 m, shrubs: 1–3 m, and trees: >3 m). Data were collected within a radius of 25 m from the set of traps.

### 2.2. Descriptive Variables

Mosaic habitat was described by the following quantitative (altitude, distance from the sea, agriculture intensity, anthropic disturbance, number of honeybee colonies, number of hornets) and qualitative (habitat type, dominant plant species) ecological and anthropic-related variables (Table S1). Agriculture intensity and anthropic disturbance were estimated on an ordinal scale ranging from one to three, where one is low, two is equal to medium, and three is high. Agriculture intensity reflects agricultural intensification estimated during field visits, while anthropic disturbance includes signs of human activities (e.g., waste or mowing and gardening activity, the proximity of a golf course) and human-related constructions such as small buildings, walls, fences, and bike trails. Three habitat types were described as herbaceous, shrubs, or trees. In each location where trees were present, the dominant tree species was also determined.

### 2.3. Sampling

Sampling was carried out during September and October 2019, every seven days, which is the late bloom period in the area and, thus, the specific fructification period. For insect collection, we used commercial traps that had been purchased at an agricultural store. The trap was a plastic container in which a wasp drowned in the attractant liquid and could not get out. A total of sixteen traps were placed on trees about 2 m above ground level and approximately 5 m from the honeybee hives. The sampling locations were on the private land of beekeepers, and we had permission to place the traps. We provided them with the results after the end of the project. On the field, we released any live species that was readily identified as our target species or was not near the place of capture. Our locations were not known for the occurrence of any taxa of conservation concern. According to standardized collection methods, we performed an active specimen orientation collection method with chemical bait classified as the B2 method [37]. Each trap was filled with the attractant liquid (a total of 50 g). The attractor liquid was composed of 190 g/kg of vinegar, 3 g/kg of 38% brandy, and 240 g/kg of food-grade liquid with an additional 1 dl of blonde beer. The traps were changed every seven days, and the samples were collected and preserved in 70% ethanol.

### 2.4. Selectivity of the Collecting Method

Recorded faunal composition depends on the collecting methods [38]. Selectivity of trapping methods resulted in the high frequency and diversity of some Diptera (Drosophilidae, Muscidae, Calliphoridae, and Anthomyiidae) and Hymenoptera (Vespidae and Apidae) families. The composition of the attractant most likely influenced the spectrum of captured species (mostly fruit-attacking species). Some dipteran families (Chironomidae, Sciaridae, Empididae, Agromyzidae, and Sphaeroceridae) usually very common in other collecting methods (Malaise traps, sweeping, colored dishes with water) were practically not found. We also did not find any wild bees or bumblebees. In the present study, the use of commercial traps resulted in a high number of species and specimens trapped, as well as several pest species and other very interesting, otherwise rare findings. This can be explained by the selectivity of the commercial traps used. Several characteristics of used traps could be responsible for the selectivity: (a) attractiveness of the bait itself (most Drosophilidae, *Aulacigaster* spp., *Suillia* spp., and *Phaonia pallida* (Fabricius 1787); (b) chance occurrence (*Platypalpus*) or of groups looking for hollows (*Drapetis*, *Culicidae*); (c) vicinity of honeybee apiaries (*Achanthiptera*, several *Fanniidae*); (d) attractiveness of decaying insects trapped (*Platystoma lugubre*, Piophilidae, several *Sarcophaga* spp.); and (e) sampling period (Hymenoptera).

### 2.5. Morphological Identification

From the total trap content, we extracted specimens of Diptera and Hymenoptera. All specimens were identified at least at the family level and, if possible, at the species level. Other specimens were assigned to higher taxonomic levels due to sample degradation. The research was carried out in two locations: the Civic Museum of Natural History of Trieste (IT) (Hymenoptera) and the Czech University of Life Sciences (Diptera). Hymenoptera specimens were identified under a stereomicroscope with direct comparison with the Stolfa Collection of Vespidae (“Vespidi 3°, n° 256” e “Vespoideae II-IB 12-ex 445”). Diptera specimens were sorted under the Nikon SMZ 1500 stereomicroscope into families and readily identifiable species and selected representatives of most families were dry-prepared and sent to the specialists for closed species identification (see section “acknowledgements”). Taxa represented by a small number of specimens were counted in full, more numerous taxa were counted from smaller subsamples (usually an eighth of a sample), and some species were confirmed without quantitative data. For the determination of new records for Croatia, the following databases were consulted: EASIN (European Alien Species Information Network), Fauna Europaea, GISD (Global Invasive Species Database), and EPPO (European and Mediterranean Plant Protection Organization). Voucher specimens are deposited in the collection of the Czech University of Life Sciences Prague (acronym: CULSP).

### 2.6. Statistical Analyses

Since there was no statistical difference between the sampling dates, we analyzed the data for each location separately, but with no temporal separation. All collected samples were pooled into one sample per location, unifying six individual samplings. Data analysis was performed only for species with counted units. Different statistical analyses were used to determine species richness and composition patterns for different response variables. Specifically, Diptera’s and Vespidae’s species richness patterns have been described using classic extrapolation sampling curves of species richness for individual-based abundance data. Bootstrap confidence intervals around the diversity for extrapolated samples have also been calculated to facilitate comparisons of diversities across multiple sites [39]. The function *iNext* in the R package *iNext* [40] was used. A correlation analysis based on the Pearson coefficient was used to evaluate the pairwise linear relationship between species richness and the set of quantitative environmental variables.

A generalized linear model (GLM) [41] was estimated to find the set of environmental variables useful to explain variability in species richness. Poisson error distribution was selected as a fitting parameter in GLM to model species richness (count data). The adequacy of the selected error distributions in GLM and the occurrence of linear relationships between responses and predictors were checked and tested on model residuals once the model was performed. The significance of each predictor in the linear predictor was tested using the X^2^ statistic. As a measure of “goodness of fit” for each GLM, the adjusted D^2^ (D^2^adj) was calculated [42]. The minimally adequate model (including only significant environmental variables) was found based on the minimization of the Akaike information criterion (AIC) considering the combination of all the subsets of environmental predictors. The function *glmulti* in the *glmulti* R package [43] was used to compare the whole set of models and to select the best-reduced one. The same modeling approach was used to model species richness for pests in relation to environmental variables. Moreover, since *Robinia pseudoacacia* is the most spread alien tree plant species in the study region, we investigated the relation to the distribution of *Callopistromia annulipes* (Macquart, 1855). To have a clearer idea of the effect of *R. pseudoacacia* on the distribution of *C. annulipes*, an independent one-tier *t*-test was carried out to test if *R. pseudoacacia* stands had a higher abundance of this pest.

The species composition of Diptera and Hymenoptera was analyzed via redundancy analysis (RDA). Specifically, the RDA was based on the Hellinger-transformed plant species abundances constrained by all the environmental predictors. Quantitative predictors were standardized (mean 0, 1 standard deviation) before running the analysis. RDA analysis and tests for statistical significance (for constrained axes and environmental predictors) were performed using the “*rda*”, “*anova.cca*”, and “*permustats*” functions within the “*vegan*” v.2.5–7 package [44]. Finally, we carried out an abundance-based indicator species analysis to detect the indicator species for different levels of anthropic disturbance, habitat type, and agriculture intensity factors. We used the function *multipatt* in the package *indicspecies* [45].

## 3. Results

### 3.1. Diptera and Vespidae Diversity

**Figure 2:**
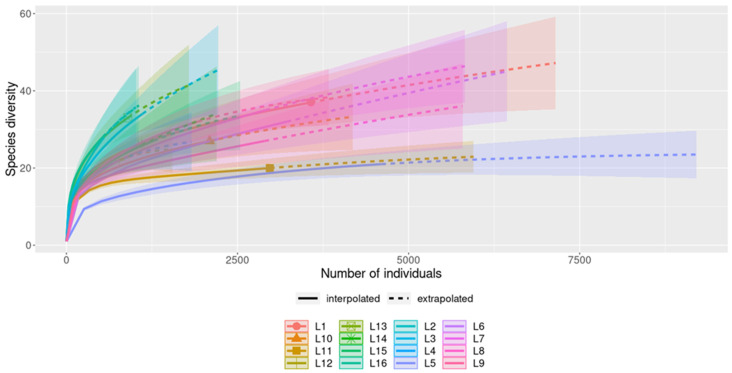
Analyses of sampling efficiency by extrapolation methods for each sampled site. Dotted lines represent extrapolated values. Individual-based randomized species accumulation curves for each sampling location are also shown (continuous lines). Symmetric 95% CIs are associated with each curve (shaded areas).

From sixteen sampling locations, we collected 80 samples. In total, we isolated 31.284 individuals belonging to 94 species and 24 families (Table S2). Total species richness varied from 20 to 37 (Table S2). A significant number of autochthonous hornets *Vespa crabro* (L., 1758)*,* a known bee predator, were trapped (Table S1). The species *V. crabro* is unable to destroy a honeybee colony like invasive hornets. Still, due to its high predation rate, especially at the end of the summer, its presence can be extremely costly, especially for weakened honeybee colonies [46]. We identified 807 specimens of *Vespa crabro* and 1.969 specimens of wasps (Vespidae family). The invasive alien hornet species *Vespa velutina* Lepeletier, 1836, and *Vespa orientalis* L. 1771. have not been found. A total of 2.776 individuals belonging to one family (Vespidae) and four species of Hymenoptera were recorded. The majority of collected wasps have been identified as *Vespula germanica* (Fabricius 1793) and *Vespula vulgaris* (Linnaeus 1758). In the traps, we also found nine specimens of the *Polistes* genus (Table S2). A total of 28,508 specimens of Diptera were found. Special attention was paid to pests and newly recorded species (details are in Section 3.2 and Section 3.3). We identified 23 families and 90 species of Diptera insects in the traps (Table S2). The most abundant family in all the investigated localities was Drosophilidae (abundance frequency: 52.6%), followed by Muscidae (20.9%) and Heleomyzidae (5.6%). Comparing accumulation curves between localities showed significantly higher total species richness in Diptera and Vespidae, indicating that some species remained undetected (Figure 2).

### 3.2. Pests Findings

*Drosophila suzukii* (Matsumura, 1931) was the most abundant invasive alien species found at every sampling site. Moreover, we found a total of 34 specimens of *Chymomyza amoena* (Loew 1862); this species was previously confirmed only twice in Croatia [30,47]. *Callopistromyia annulipes* was also found in fourteen locations with 265 specimens, representing the second record in Croatia. *Atherigona varia* (Meigen, 1826) (89 specimens), *Bactrocera oleae* (Rossi, 1790), (27 specimens), *Silba adipata* (McAlpine 1956) (15 specimens) and *Ceratitis capitata* (Wiedemann 1824) (1 specimen) were also discovered in collected samples. More information on the most relevant pest species is presented in Table 1.

### 3.3. First Findings

Seventeen species have been recorded for the first time in Croatia (Table 2).

### 3.4. Correlation with Environmental Variables

**Figure 3:**
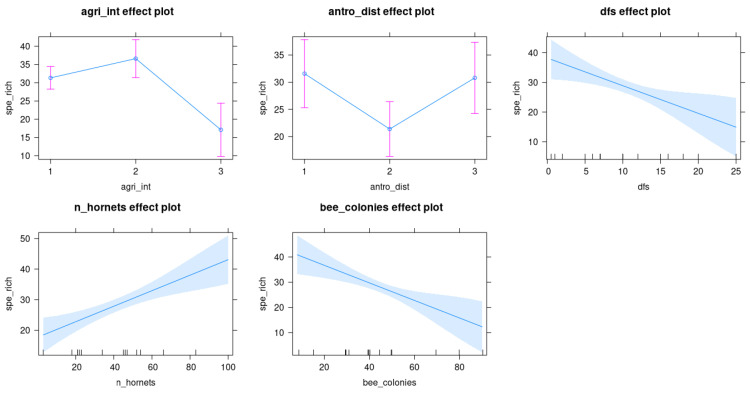
Effect plots based on the estimated coefficients for environmental variables (agriculture intensity, anthropic disturbance, distance from the sea, number of hornets, and number of honeybee colonies) retained in the minimally adequate model of total insect species richness (Poisson GLM with log link function).

A description of the habitats is presented in Table S1. The correlation plot showed that distance from the sea and the number of honeybee colonies were negatively correlated to total species richness (Table 3 and Figure 3). The number of hornets was positively correlated to species richness (Table 3 and Figure 3).

Effect plots based on model-estimated coefficients explained the relationship between total species richness and three ecological variables (distance from the sea, number of hornets, and number of honeybee colonies) and two anthropic variables (agriculture intensity, anthropic disturbance) included in the minimal adequate model (Figure 3). Total species richness increased with low to medium agriculture intensity, although it was not statistically significant. The species richness of Diptera varied between 20 and 37 species per location. It was noted that the highest level of anthropic disturbance was present in 5 out of 6 locations with species richness above 30.

**Figure 4:**
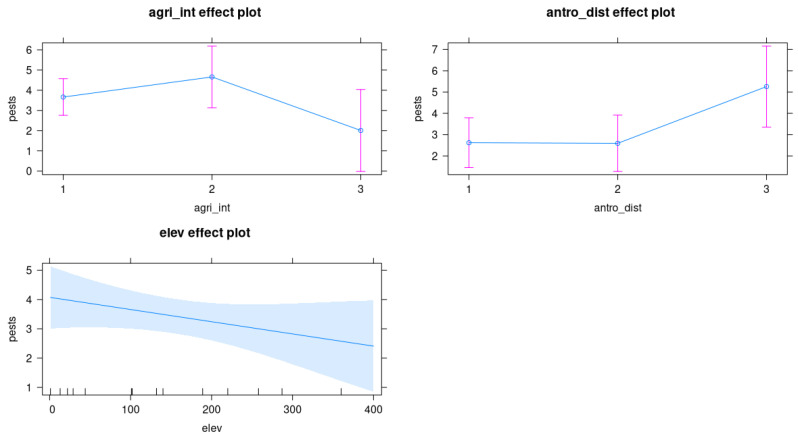
Effect plots based on the estimated coefficients for environmental variables (agriculture intensity, anthropic disturbance, and elevation) retained in the minimally adequate model of pest species richness (Poisson GLM with log link function).

The number of pest species is also negatively correlated to elevation and distance from the sea (Figure 4 and Figure S1). Generalized linear models for pest species richness showed that anthropic disturbance is positively correlated with pest species richness (Table 4).

**Figure 5:**
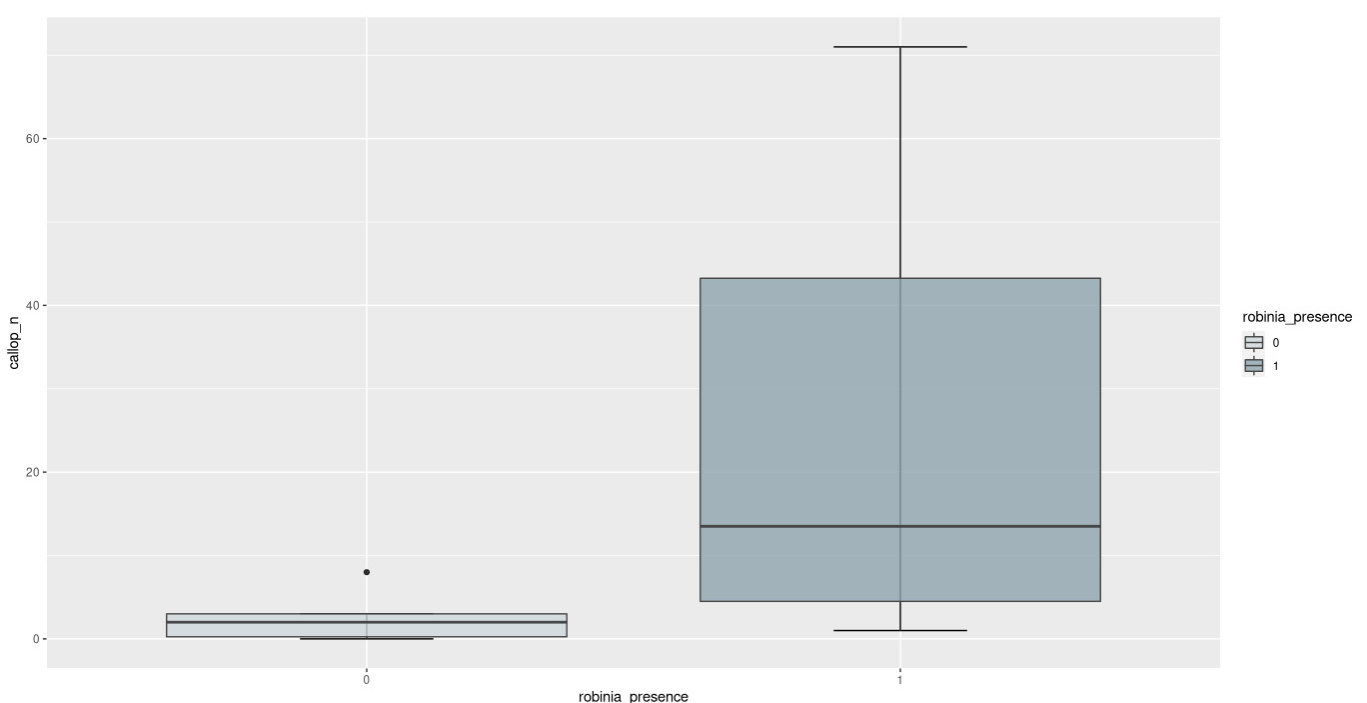
Correlation between dominant plant species *Robinia pseudoacacia* and pest *C. annulipes* (1 *=* stands with *R. pseudoacacia*; 0 = stands without *R. pseudoacacia)*.

Effect plots based on model-estimated coefficients explained the relationship between pest species richness and three ecological and anthropic variables. Total pest richness increased in the location with high anthropic disturbance, although it was not statistically significant (Figure 5). The analysis confirmed a significant (positive) influence (t = -2.67, df = 9.44, *p*-value = 0.012) of *Robinia pseudoacacia* stands on the presence of *Callopistromyia annulipes*.

**Figure 6:**
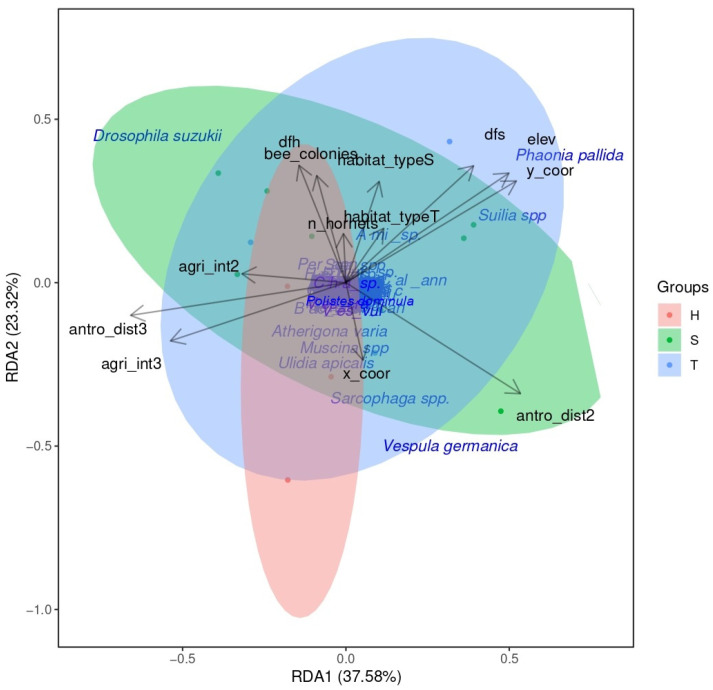
RDA analysis of species composition in relation to environmental and anthropic predictors. Note: categories of habitat type where H is herbaceous, S is shrubs, and T is trees. Predictors abbreviations: dfs—distance from the sea; elev—elevation; agri_int—agriculture intensity; antro_dist—anthropic disturbance.

The first two axes of the RDA (see the biplot in Figure 6) explained 60.9% of the total variation in species composition, where the first axis explained 37.58% and the second 23.32%. Specifically, the first axis highlighted a gradient of increasing agricultural and human impact intensity (from right to left) that inversely moves with the distance from the sea and with the altitudinal gradient (from left to the right). Along with these two inverse gradients, species such as *Phaonia pallida*, *Suilia* spp., *Atherigona varia,* and *Ulidia apicalis* (Wiedemann, 1824) differed. The biplot identified one group where the highest number of *D. suzukii* was found. On the right, three locations with an elevation higher than 200 m and a distance from the house higher than 100 m showed the highest abundance of the species *Phaonia pallida.* The second axis, on the other hand, was positively related to the number of honeybee colonies and the number of hornets connected with the T and S habitats.

Indicator species analysis (Table 5) showed that *Bactrocera oleae* and *Scathophaga stercoraria* are found in all locations with high agriculture intensity. Additionally, *A. varia* was found in all locations with medium to high agriculture intensity. *B. oleae* was also found in all locations with the highest anthropic disturbance. *Bercaea africa* (Wiedemann, 1824) was found in all locations with the habitat type herbaceous. *Chymomyza amoena* was found in all locations with the shrub habitat type.

## 4. Discussion

The present work assesses the Diptera biodiversity of captured taxa in a mosaic agricultural landscape in the proximity of apiaries and in the presence of insect pests. At the same time, it investigates dependency on environmental and anthropic variables. Our case study may well reflect a situation typical for other Eastern and Mediterranean European countries whose agricultural landscapes are still structurally complex and rich in biodiversity [83].

### 4.1. Pests Findings

Globalization and the increased movement of people led to the rapid spread of pests into new habitats [84]. The ongoing flow of plant pests between countries has been recorded in Europe [85], the USA [86], The Netherlands [87], Japan, and Australia [88,89]. The majority of the introductions of alien insects in Europe are associated with the international trade in ornamental plants. Alien species often cause enormous costs to agriculture, forestry, and human health [90,91,92]. Since the increased spread and worldwide distribution of plant pests, fast detection is essential for effective control and management measures. This study gives special attention to searching for the yellow-legged hornet *Vespa velutina*, an important invasive species causing environmental and economic damage in new areas. Fortunately, the Asian yellow-legged hornet was not detected.

Some of the species reported within this study are important economic pests since they cause severe damage to agricultural production [93,94,95]. Some of them are being recorded in Croatia for the first time. There is no up-to-date species list of invertebrates harmful to plants recently established in Croatia. In the Istria region, Diptera plant pests have never been systematically researched. So far, only a few families have been investigated in this region: Drosophilidae, Sarcophagidae, Muscidae, Tephritidae, and Culicidae. The research on species from Drosophilidae and Tephritidae has been focused mainly on the most common pest species, such as *Drosophila suzukii* and *Ceratitis capitata* [96,97]. Literature records were summarized and have shown the presence of 148 species from the Sarcophagidae family in Croatia [98]. We found twenty-four species from the family Sarcophagidae, of which fifteen were also detected in the study of Krčmar et al. (2019) [98]. More recent research in Istria [99,100] reports a list of 97 known species from the Muscidae family in Croatia, of which twenty-two were found in this study. The study by Merdic et al. (2008) [101] on the Culicidae family has shown a great faunistic diversity (54% of the total Croatian mosquito fauna) with twenty-seven determined mosquito species. The recent faunistic data by Verves and Bartak (2021) comprises a list of thirty-three species of Sarcophagidae in Croatia [102], some also mentioned in this study.

In our study, we particularly paid attention to seven main agricultural and forest pest species. All of the found species, besides *Callopistromyia annulipes*, are registered in the EPPO Global Database as important pests. *Drosophila suzukii* (Matsumura, 1931) (17.352 specimens) was found in all sixteen locations in our study in 80 samples. This species has since 2010 spread on new cultivated and wild host plants, causing damage to raspberries, peaches, and grapes [103,104]. *D. suzukii* was included in the EPPO A2 list of pests recommended for regulation as a quarantine pest in 2011. This species was found at every investigated locality, regardless of altitude or habitat type. The Istrian region is well known for its high production of wine grapes (*Vitis vinifera* L.) and figs (*Ficus carica* L.) in Croatia. During the late summer and early autumn months, those fruits are in full production, providing food, shelter, and suitable hosts for the reproduction of *D. suzukii.* Recent studies confirm that *D. suzukii* in European habitats is an important economic pest in fruit production since it causes severe damage to strawberry, apple, pear, grape, and fig production [105,106,107]. Even though *D. suzukii* is well known as a threat to fruit production, the investigation of its influence on grapes and viticulture has only recently begun [108]. The authors of this study concluded that, at present, *D. suzukii* might not be considered a threat to viticulture in North Italy. Still, further studies are needed to better understand the relationship between *D. suzukii* and grapevine. On the other hand, some other invasive and dangerous species from the Drosophilidae family (*Zaprionus* spp.) were not captured in the Istria region in Croatia. Possibly they have not invaded this area so far. Apart from *D. suzukii*, other species from the same family can also breed in various fruits [96].

The pest fly *Callopistromyia annulipes* was found for the second time in Croatia during our study [57]. This species originally had a Nearctic distribution but has been discovered in several European countries since 2007: Slovakia [55], France [56], Belgium [109], and Germany [54]. This species, native to North America, has rapidly spread through Europe since its first appearance in Switzerland in 2007, recorded by Merz (2008) [72]. It is usually found near *Robinia pseudoacacia* and *Acer negundo* trees or trunks [56]. The high number of individuals found in this study could be related to the dense population of *R. pseudoacacia* in the Istrian region (OIKON). In this study, we statistically proved the relationship between *C. annulipes* and *R. pseudoacacia* (Figure 5). In the few studies that have been carried out up to date, no known harmful behavior of this species in Europe has yet been observed [110].

The presence of the species of sorghum shoot fly, *Atherigona varia*, has been recorded. *A. varia* is one of the most important grain sorghum pests in Asia, Africa, and Mediterranean Europe [58,111]. It causes damage to the seedlings from 1 week to 30 days of age. The typical symptom of damage is the drying of the central shoot, called the “dead heart.” The common appearance of this species could be correlated with the large population of wild *Sorghum* species in the Istria region (https://invazivnevrste.haop.hr/katalog/5647, accessed on 20 October 2022). *Sorghum* species spread is connected to agriculture and causes severe impacts [112], so we proved the link between *A. varia* and agriculture intensity. In fact, *A. varia* was found at all locations with medium-to-high agriculture intensity.

The presence of *Chymomyza amoena* has been confirmed in the northern part of Croatia [30], and this study has revealed its presence at six new localities in the Istria region. The collecting localities are surrounded by Mediterranean forests and extensive production of grapes, figs, and sweet cherries. So far, this species has been recorded throughout Europe [113,114], but its occurrence is “pointed” in a particular area [115]. Although it is considered an invasive alien species in Europe, the abundance *of C. amoena* within the natural population is small and, so far, does not represent a threat to fruit production.

In the samples, we have found the olive fruit fly *Bactrocera oleae*, one of the most important olive pests of the Mediterranean basin. Accordingly, this study found it in all locations with high anthropic disturbance and intensity. This pest occurs every year, and its success is mainly controlled by climate conditions (temperature, rainfall, and relative humidity) [116]. For example, in addition to causing significant losses to nature, the infection of the olive fly contributes a lot to the decline in oil quality [117]. Consequently, this species is responsible for high economic losses in olive oil production [118].

The species *Silba adipata* found in our samples can cause significant economic damage to fig fruits by infesting unripe figs and leading to premature fruit drop [119]. In the last two decades, a high intensity of infection has been recorded in coastal Croatia and along the entire coastal region of Croatia [65].

We found the Mediterranean fruit fly, *Ceratitis capitata*, in only one sample. The Mediterranean fruit fly is one of the world’s most destructive fruit pests and is widely distributed all over the world (https://www.cabi.org/isc/datasheet/12367, accessed on 5 October 2022). It has a high economic impact, affecting production, control costs, and market access. *C. capitata* has been present in Croatia for more than 70 years [120]. Since then, it has become an important pest, particularly in citrus production, but also in apple, peach, apricot, pear, and grape production [121]. To prevent the spread of this species in Croatia, the Ministry of Agriculture published an action plan with flat measures for eradication in 2018. The symptoms of fruit infestation and size of *C. capitata* larvae are similar to those of Black fig fly, *Silba adipata,* and the damages are often mistaken.

### 4.2. First Records in Croatia

In this study, seventeen species have been recorded in Croatia for the first time, which is an important number of additions to the Croatian insect fauna. *Ulidia apicalis* Meiden, 1826, is a species from the Ulidiidae family so far confirmed in France (south, mainland, Corsica), Italy (Sicily), Portugal, Spain, Morocco, and Tunisia [122]. More recent distribution data show its presence in Greece and Turkey (https://www.inaturalist.org/taxa/322843-Ulidia-apicalis, accessed on 3 November 2022). In this study, *U. apicalis* was found in all locations with medium-to-high agricultural intensity, which may be the reason for its spread in the Mediterranean. Another species new to Croatia from the Ulidiidae family is *Herina lacustris* Meigen. It is a Western Palaearctic species [72].

We found *Desmometopa discipalpis* (Papp, 1993), a species with limited records available. According to records from Roháček 2016, *D. discipalpis* is a saprophagous thermophilous species inhabiting rotten wood and/or excrement [83]. Another species of the genus *Desmometopa, D. microps*, was found. This species, originally distributed in the Oriental, Afrotropical, and southeastern Palaearctic regions, expanded into Central Europe approximately 10 years ago [123]. Our records show further spreading of *D. microps*.

The captured *Periscelis piricercus* is important because it represents the new easternmost distribution limit of the species, which is otherwise only known from Portugal, Spain [88], and Switzerland [124].

*Periscelis winnertzii* Egger, 1862 (=*P. fugax* Roháček and Andrade, 2017), see Roháček (2022) [125], which was described and illustrated based on a series of specimens from Portugal and the Czech Republic [78]. This is the first record from Croatia, represented by two specimens. In our samples, we also recorded a high number of other rare groups from the *Periscelis* genus. We also found *Toxoneura muliebris* (Harris, 1780), known as the flutter fly because the males extend and vibrate their wings. According to a previously published report [126], flutter fly larvae can be saprophagous, phytophagous, or carnivorous. *T. muliebris* of the Pallopteridae family ranges in Europe from Spain and Italy to Great Britain, France, Austria, Turkey, Ireland, and western Russia [127].

*Cephalia rufipes* (Meigen, 1826) is a species with a northern Mediterranean and central European distribution, occurring from the Iberian Peninsula, southern Germany, Austria, Israel, and Portugal [80]. This work shows that its geographical distribution has broadened.

An extremely rare species, *Phaonia regalis* (Stein, 1900), was recorded. So far, it has been found in Austria, Bulgaria, Greece (Cyclades Islands), Georgia, and Turkey (https://fauna-eu.org/cdm_dataportal/taxon/ac3f7319-3974-4cc1-859d-a9e916aaa397, accessed on 7 November 2022) [128]). *Grzegorzekia hungarica* is also a new species in Croatia; so far, it has been recorded only in Hungary [74] and Slovakia [129].

Recording the first appearance of species in different habitats is important for distribution tracking and taking timely management or removal actions. Our data provide information on the spread and distribution of some rare Diptera species in Europe. The faunistic value of a given area can be determined mainly by the number of recorded rare species, but these species do not have any significant effect on their ecosystem services [130]. These authors found that rare species in Poland were more abundant in natural habitats, while common species dominated farmlands. Our study also shows that human-dominated habitats can sink rare and new species.

### 4.3. Species Diversity in the Mosaic Agricultural Landscape and Correlations with Different Variables

Generally, agriculture and anthropic influence are major drivers of biodiversity loss [131]. Complex landscape structures specific to intensive agriculture and increased use of agrochemicals are the main drivers of the reduction of arthropod species richness [132,133,134]. Some authors have proved that anthropogenic influence in specific habitats linearly decreased the Diptera diversity [135]. On the other side, much biodiversity can be retained within specific agricultural landscapes [136]. Land use changes in the last decade included intensification of land use in some areas and land abandonment in others. Although the loss and fragmentation of semi-natural and mosaic habitats are prominent causes of biodiversity loss, little is known about the species diversity in different agricultural landscapes [22]. Like in many other Eastern European countries, traditional practices have created small-scale mosaic landscapes. For example, in Croatia, the average size of an agricultural farm was 0.51 ha [17]. Our study may well reflect a situation that is typical for many Eastern European countries whose agricultural landscapes are still considered biodiversity hotspots [84]. Previous studies have suggested that habitat type is an important factor in explaining the diversity of insects [137,138]. It is well understood that agricultural practices, particularly conventional farming, have a direct or indirect impact on pollinator populations [139].

This investigation provides data on Diptera and Vespidae species richness across gradients of agriculture and anthropic influence and in response to variables representing altitude, vegetation type, honeybee colony number, and the number of hornets. Our research showed that mosaic agroecosystem areas in Istria still sustain a significant number of Diptera and Vespidae species. As expected, anthropic influence positively correlated to pest richness since these species depend on human activities.

It is known that polycultures and florally diverse environments support native pollinator diversity due to a continuous supply of food resources [140,141]. Casanelles-Abella and Moretti (2022) proved that apiaries in cities are a potential problem for the biodiversity of some pollinator species, especially wild bees [142]. In fact, maybe for this reason, we did not capture any wild bees in our traps. In our study, the relationship between the size of the apiaries and species richness was statistically confirmed. The number of honeybee colonies was negatively correlated to species richness. Honeybees can have a negative impact on wild pollinators [143]. The study by Torné-Noguera et al. (2015) supports the hypothesis that high honeybee apiary densities may have an impact on other insect pollinators via competition for flower resources [144]. This is in accordance with our results showing that a higher density of honeybee hives has a lower total species richness and fewer rare first findings. Moreover, a complete absence of wild bees in the traps could also be connected to the presence of apiaries and the result of food competition with honeybees.

We found the lowest species richness in locations L5 and L11, characterized by low agricultural intensity and low anthropic influence. Moreover, our study recorded a positive correlation in insect richness and composition between environments with lower to higher agricultural influence and between environments with different anthropic influences. This appears to be in concordance with the results documented in similar analyses on Hymenoptera diversity, where insect richness was positively connected to some extent of human activities. Semi-natural habitats providing different feeding and nesting resources proved to be diversity hotspots [138]. We assume that high insect biodiversity in our study area results from agricultural practices still dominated by semi-intensive farming and labor-intensive, traditional techniques with low levels of agrochemical inputs.

The agricultural intensity was not clearly related to species richness, and it was not possible to prove statistically any correlation between those two factors. We observed that both species richness and the number of pests were statistically (negatively) associated with elevation and distance from the sea. Other authors have also confirmed the connection between species richness and those two elements. Martín-Vega et al. (2016) found that elevation is negatively correlated with species richness in Spain [145]. It can be assumed that this is connected with the transport of people and goods along the coastline. Mosaic semi-natural landscapes support high plant species diversity [146] and consequently also high insect diversity [141]. Proper management of artificial nests for solitary bees in semi-natural mosaic agricultural areas can support and maintain pollination services [147], but only after limiting pesticide use and planting native flowering plants between crop fields (prairie strips).

## 5. Conclusions

The inventory performed in the mosaic agricultural landscape in the Istrian Region (western Croatia) gives new information on the diversity of Diptera and Vespidae in the Mediterranean. Selectivity of the trapping method resulted in a high frequency of some Diptera (Drosophilidae, Muscidae, Calliphoridae, and Anthomyiidae) and Vespidae species and the absence of otherwise frequent species in a similar environment (Chironomidae, Sciaridae, Empididae, Agromyzidae, and Sphaeroceridae). The number of honeybee colonies was negatively correlated to species richness. Pest richness was positively correlated with anthropic influence since these species depend on human activities. The agricultural intensity was not clearly related to species richness, and it was not possible to prove statistically any correlation between those two factors. Assessing pest spread is important for generating a new understanding of environmentally friendly agroecosystems and directing management actions. Our results can be helpful for decision-makers and local authorities to develop appropriate conservation strategies and monitoring measures for pests to preserve biodiversity within Mediterranean mosaic agroecosystems. Several Diptera were recorded for the first time in Croatia, providing new information on the distribution of some rare species in South Europe. Our data suggest that mosaic agricultural landscape contributes to Diptera and Vespidae diversity in semi-rural landscapes. That leads us to the conclusion that it is necessary to diversify the types of semi-natural habitats to promote a variety of plant communities and natural nesting sites. Since the high specificity of semi-intensive agriculture landscapes, the relation between habitat specificity and habitat-specific insect communities should be explored in the future, as well as their contribution to insect diversity.

## Figures and Tables

**Table 1:** List of agricultural and forest Diptera pests, their distribution, and host plants; * invasive assumes alien invasive species.

Species/ Family	Number of Specimens	Location	Invasive * and Pest Status in Croatia	Pest Status Worldwide/References	Host Plants
*Drosophila suzukii* Drosophilidae	17,352	L1-L16	Important pest/invasive	Worldwide pest; Italy [48], Spain [49], Poland [50], USA [51], Argentina [52], and India [53].	fruits vegetables
*Callopistromyia annulipes* Ulidiidae	265	L1-L11, L13, L15, L16	n.d., the second record, invasive	Pest in Europe: Germany [54], Slovakia [55], France [56], and Croatia [57].	deciduous dead trees
*Atherigona varia* Muscidae	89	L1, L3, L5-L9, L13, L16	Important pest/invasive	Pest in Europe and Asia: Turkey [58] and India [59]	corn, sorghum
*Chymomyza amoena* Drosophilidae	34	L1, L2, L6, L8, L9, L15	Important pest, invasive, third appearance in Croatia	Pest in Europe; Croatia [30], The Netherlands [60], and Switzerland [61].	fruits, nuts
*Bactrocera oleae* Tephritidae	27	L1, L3, L6, L7, L9, L14, L15	Important pest/domestic	Worldwide pest: Greece [62], Pakistan, India, Nepal [63], Kenya, Tanzania, Zanzibar, Uganda, and DR Congo [64].	olive trees, fruit
*Silba adipata* Lonchaeidae	15	L7	Important pest	Pest in the Mediterranean, South Africa: Croatia [65], Tunisia [66], and South Africa [67].	fruit
*Ceratitis capitata* Tephritidae	1	L7	Important pest	Worldwide pest: Morocco [68], Turkey [69], and South African Republic [70].	fruit, vegetable, nuts

**Table 2:** First record species for Croatia and their distribution in Europe.

Species/ Family	Number of Specimens	Location	Other Records Inside Europe/References	Host Plant
*Ulidia apicalis* (Wiedemann, 1824) Ulidiidae	464	L1, L2, L3, L4, L5, L6, L7, L8, L9, L10, L11, L12, L13. L14, L15, L16	Italy Spain, and Portugal [71]	possibly flowers
*Herina lacustris* (Meigen, 1826) Ulidiidae	58	L1	Spain [72] and France [71]	n.d.
*Desmometopa microps* (Lamb 1914) Milichiidae	4	L7, L10, L12	Czech Republic and The Netherlands [71,73]	n.d.
*Periscelis (Myodris) piricercus* (Carles-Tolrá & Verdugo Páez, 2009) Periscelididae	3	L9, L10	Spain [74] and Portugal [75]	trees
*Toxoneura muliebris* (Harris, 1780 Pallopteridae	3	L4, L15	Ireland [76]; Russia [77], Italy France, Spain, The Netherlands, Greece, and Portugal [71]	possibly saprophagous species, flowers
*Periscelis (P.) winnertzii* (Egger, 1862) Periscelididae	2	L10, L11	Portugal and Czech Republic [78], Finland, France, and UK [71]	n.d.
*Cephalia rufipes* (Meigen, 1826) Ulidiidae	1	L4	Spain [79], Portugal [80], France, Germany, The Netherlands, and Austria [71]	n.d.
*Desmometopa discipalpis (Papp, 1993)* Milichiidae	1	L2	Greece [81], Germany [82], Czech Republic [83], Sweden, and Denmark [71]	n.d. (possibly saprophagous species)
*Phaonia regalis* (Stein, 1900) Phaonia	/	/	Austria and Bulgaria [71]	n.d.
*Grzegorzekia hungarica* (Papp & Ševčík, 2007) Mycetophilidae	/	/	Hungary and Romania [84,85]	n.d.
*Lonchaea peregrina* (Becker, 1895) Lonchaeidae	/	/		
*Lamprolonchaea smaragdi* (Walker, 1849) Lonchaeidae	/	/	Spain, Portugal, and Greece [71,86]	vegetables, crops
*Neoalticomerus formosus* (Loew, 1844) Odiniidae	/	/	The Netherlands [87], Sweden, Finland [71], Poland, France, and Italy [87]	n.d.
*Odinia ornata* (Zetterstedt, 1838) Odiniidae	/	/	Sweden, Finland, and UK [71]	n.d.
*Amiota alboguttata* (Wahlberg, 1839) Drosophilidae	/	/	Sweden, Finland, UK, and Norway [71]	possibly fermenting tree sap
*Scaptodrosophila deflexa* (Duda, 1924) Drosophilidae	/	/	UK, Sweden, Finland, Switzerland, and The Netherlands [71,88]	n.d.

**Table 3:** Generalized linear model for total species richness. Statistical significance: ** *p* < 0.05; * *p* < 0.01.

Selected Variables	Degree of Freedom	Sum Sq	Coefficient Sign	*p*-Value (F Tests)
Agriculture Intensity	2	59.43	Factor	0.071
Anthropic Disturbance	2	98.08	Factor	0.025 *
Distance from the sea	1	4.62	−0.932	0.458
N° of hornets	1	36.16	0.253	0.064
N° of bee colonies	1	112.66	−0.348	0.006 **
R-squared	0.855 **			

**Table 4:** Generalized linear model for pest species richness. Statistical significance: ** *p* < 0.05; * *p* < 0.01.

Selected Variables	Degree of Freedom	Sum Sq	Coefficient Sign	*p*-Value (F Tests)
Agriculture Intensity	2	4.52	Factor	0.168
Anthropic Disturbance	2	10.20	Factor	0.034 *
Elevation	1	2.63	−0.004	0.145
R-squared	0.621 **			

**Table 5:** Indicator species analysis. Statistical significance: ** *p* < 0.05; * *p* < 0.01.

**Agriculture Intensity—level 3**					
Species	A	B	stat	*p*-value	significance
*Bactrocera oleae*	0.7835	1	0.885	0.0186	*
*Scathophaga stercoraria*	0.8082	0.75	0.779	0.0224	*
**Agriculture Intensity—combined levels 2 and 3**					
	A	B	stat	*p*-value	significance
*Atherigona varia*	0.8992	1	0.948	0.0038	**
**Anthropic disturbance—level 3**					
	A	B	stat	*p*-value	significance
*Bactrocera oleae*	0.807	1	0.898	0.0038	**
**Habitat type—habitat H (herbaceous)**					
	A	B	stat	*p*-value	significance
*Bercaea africa*	0.7903	1	0.889	0.0098	**
*Heteronychia filia*	1	0.6667	0.816	0.021	*
**Habitat type—habitat S (shrubs)**					
	A	B	stat	*p*-value	significance
*Chymomyza amoena*	1	0.6667	0.816	0.4446	*

## Data Availability

Data are presented in the manuscript.

## Supplementary Materials

The following supporting information can be downloaded at: https://www.mdpi.com/article/10.3390/ani13061024/s1, Supplement Table S1. A detailed description of locations (geographical, anthropic, ecological data). Geographical, anthropic, and ecological variables of selected sites and the number of collected hornets (*Vespa crabro*) as the main bee predator (Agriculture diversity: 1—low, 2—medium, 3—high diversity; Anthropic disturbance: 1—low, 2—medium, 3—high disturbance). Supplement Table S2. All recorded Diptera and Hymenoptera species and families for each location. Locations (L) correspond to sites described in Table 1 and Figure 1. Supplementary Figure S1. The correlation of ecological variables (DFS: distance from the sea, elevation, and number of pests) with pest richness. The blue gradient expresses positive correlation coefficients, and the red gradient expresses negative coefficients. Statistical significance: *** *p* < 0.001; ** *p* < 0.05; * *p* < 0.01.
