# Supplementary material for: The Impact of Beehive Proximity, Human Activity and Agricultural Intensity on Diptera Diversity in a Mediterranean Mosaic of Agroecosystems, with a Focus on Pest Species
Source: Animals (Basel). 2023 Mar 10;13(6):1024. doi: 10.3390/ani13061024 (PMC10044344; doi:10.3390/ani13061024)
Supplement: Supplementary file 1 [file animals-13-01024-s001.zip › Figure S1.pdf]

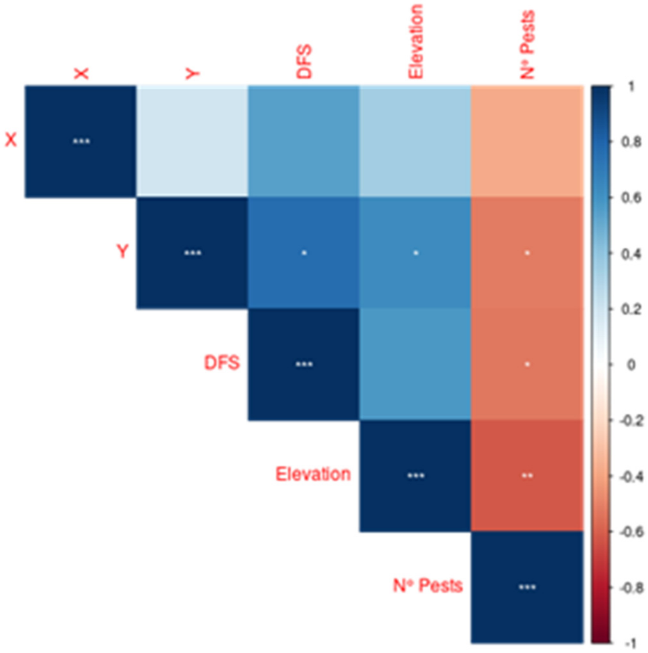

Supplement Figure S1. The correlation of ecological variables (DFS: distance from the sea, elevation and number of pests) with the pest richness. Blue gradient expresses positive correlation coefficients while red negative coefficients. Statistical significance: *** p<0.001; ** p<0.05; * p<0.01.
